# Sexual violence against female sex workers in The Gambia: a cross-sectional examination of the associations between victimization and reproductive, sexual and mental health

**DOI:** 10.1186/s12889-015-1583-y

**Published:** 2015-03-19

**Authors:** Jennifer A Sherwood, Ashley Grosso, Michele R Decker, Sarah Peitzmeier, Erin Papworth, Daouda Diouf, Fatou Maria Drame, Nuha Ceesay, Stefan Baral

**Affiliations:** Johns Hopkins School of Public Health, 615 N Wolfe St. #5041, Baltimore, MD 21205 USA; Enda Santé Senegal, 56, Comico VDN, BP 3370 Dakar, Sénégal; University of Gaston Berger, P.O. Box 17470, St Louis, Sénégal; Lilunga House, Fifth Floor, Somhlolo Street, Mbabane, Swaziland

**Keywords:** Female sex workers, The Gambia, Forced sex, Violence, HIV/STIs, Reproductive health

## Abstract

**Background:**

Female sex workers (FSW) are a vulnerable population for sexual violence and poor sexual and reproductive health outcomes. Sexual violence against FSW has not been widely studied in The Gambia. This study will report the prevalence of and evaluate the health issues correlated with forced sex perpetrated by clients against FSW in The Gambia, and will secondly aim to inform future research and efforts to improve health outcomes for survivors of violence.

**Methods:**

A cross-sectional survey was administered among 251 FSW accrued through a combination of chain referral and venue-based sampling in The Gambia. Eligibility criteria included being over 16 years old and having exchanged sex for money, goods, or favors in the past 12 months.

**Results:**

There is a high prevalence of sexual violence against FSW in The Gambia, with 29% (n = 70) of participants reporting a client forced them to have sex in their lifetime. Women who reported forced sex by a client were more likely to report symptoms of depression (aOR 2.15, CI: 1.10 – 4.16 p < 0.05), unwanted pregnancy (aOR: 2.69, CI: 1.12 – 6.49 p < 0.05) and report “no”, “difficult” or “somewhat difficult” access to condoms (aOR: 3.31, CI: 1.76 – 6.26 p < .01) compared to women who did not report forced sex. Client-perpetrated forced sex was also negatively associated with receiving any sexually transmitted infection (STI) test in the past 12 months (aOR: 0.49, CI: .26 – .91 p < .05).

**Conclusion:**

FSW who experience sexual violence by a client are more likely to experience poor sexual, reproductive and mental health outcomes. Responding to sexual violence among FSW, including providing survivors with access to post-exposure prophylaxis, emergency contraception, and mental health services, must be a priority given the prevalence of forced sex and links with poor health outcomes. Efforts to reduce sexual violence against FSW is a vital strategy to improve the health and safety of FSW as well as impact the spread of HIV/STIs in The Gambia.

## Background

Female sex workers (FSW) are among the most vulnerable populations for the acquisition and transmission of both HIV and STIs. In many parts of sub-Saharan Africa such as Guinea, Benin, and Senegal the national HIV prevalence is reported as 1.7% (2010), 1.5% (2007) and 1.0% (2010) compared to 36.7%, 40.9%, and 19.9% among FSW (OR 33.1, 44.2 and 23.7 respectively) [1-6]. In The Gambia, the most recent HIV prevalence data report a prevalence of 15.9% (2011) [7] and 35.1% (1993) [8] among FSW, compared to 1.7% in the general population [9]. Among the factors that increase FSWs’ risk of HIV is the high rate of sexual violence that threatens women’s ability to negotiate condoms [10-13] or engage in HIV risk-reduction behaviors [11,13,14]. The estimated prevalence of sexual violence victimization among FSW ranges from 33%-72% globally [15-17]. Data specifically from African contexts, for instance, Kenya (43%) [18], Namibia (72%) [17], and Rwanda (37%), [19] also report a high prevalence of victimization.

Sexual violence victimization is a likely barrier to effective STI/HIV prevention among FSW [20] and women in the general population [21]. Multiple studies have demonstrated that sexual violence against FSW has been associated with decreased condom use [11,12,22,23], condom failure [24], HIV prevalence [13,25], and other STI symptoms [24]. Women are put at direct risk of HIV/STIs during forced sex due to condom non-use and the increased likelihood for perpetrators of forced sex to be HIV-infected [20,26-29]. Forced sex can also increase indirect risk for HIV by influencing future patterns of sexual risk taking including decreased condom use and increased substance use during sex [20,26]. Finally, forced sex has been associated with increased stigma and social vulnerability as well as decreased care-seeking for health issues, which may influence future HIV and STI care [30]. Female sex workers are subject to abuse by many types of perpetrators including police, pimps, partners and clients. While some reports do not specify type of perpetrator in data collection, where assessed, clients are often the predominant perpetrator of sexual violence [13,23,31].

FSW have been shown to be at high risk for poor reproductive health outcomes including unintended pregnancy. Research examining the contraceptive needs of FSW in Madagascar reported that 52% of participants had an unintended pregnancy and 45% reported having an induced abortion [32]. Similar results were found among FSW in Kenya where 52% reported an unintended pregnancy over their lifetime and 32% reported an induced abortion [33]. While induced abortion is not in itself a poor health outcome, it is a valuable marker of unintended pregnancy. In settings where access to abortion is illegal or restricted, abortions more commonly take place in unsafe environments that can lead to serious negative health consequences [34,35]. Among the factors contributing to these poor reproductive health outcomes is the high prevalence of sexual violence among FSW, which has been associated with non-condom use [22,23] and has been given as a common reason for seeking an abortion [36,37].

Forced sex has also been found to be associated with depression among FSW, although the link has not been widely studied. The association between intimate partner violence (IPV) and depression has been more extensively explored. A range of poor mental health outcomes are associated with IPV in diverse samples of women not limited to FSW, including depression, anxiety, and post-traumatic stress disorder [38-41]. There have been few FSW-specific studies examining mental health outcomes associated with forced sex, but these include a study among 624 FSW in Mexico, where 86% of participants screened positive for depression, and victims of forced sex during sex work were significantly more likely to screen positive than those who had not experienced forced sex (OR 2.65 CI: 1.39 – 3.90 p < .001) [25]. A Nepalese study among 210 FSW found similarly high reports of depression (82%) among participants; however, victims of forced sex were not significantly more likely to suffer from depression than non-victims (80% vs. 82% respectively) [42].

Health outcomes for FSW in The Gambia including STIs, unintended pregnancy, abortion and depression have not been widely studied. In The Gambia, sex work is officially illegal [43]. Therapeutic abortions are also illegal, except in situations where it is necessary to save a women’s life [44]. Little is known about sexual violence victimization, or its associations with health outcomes, among Gambian women in the general population or among FSW. Bolstering the body of research on sexual violence in The Gambia could be useful in the production of national guidelines for a response to violence and in improving FSW and women’s health more generally. The analyses presented here contribute to filling this gap by reporting the prevalence of violence victimization among FSW as well as examining the health outcomes associated with forced sex for these women.

## Methods

### Participants/collection methods

Inclusion criteria for participants included being born a woman, reporting having sold sex for money, goods, or favors in the past 12 months, being over the age of 16, residing in The Gambia, and being able to provide verbal consent in English, Wolof or Mandinka. Participants were recruited for a cross-sectional survey between July and August of 2011 through a combination of peer-based, chain-referral, and convenience sampling at sex work venues. The initial participants were members of community-based organizations in The Gambia and varied in age, location of birth, education levels, and self-reported HIV status. Participants were then recruited based on peer referral as well as recruited by study staff from known sex work venues in the Greater Banjul Area and the smaller towns of Barra, Farafenni, Basse and Soma [7]. Diverse seed selection and recruitment in different regions of The Gambia were used to increase the representativeness of this non-probability sample. Sample size calculations were based on an estimated 20% HIV prevalence among FSW in Senegal, which requires a minimum of 175 participants to detect a significant difference (OR 2.0) between participants with differing HIV protective behaviors (such as consistent condom use).

Ultimately, recruitment generated a total of 251 participants who provided informed consent to participate in the study. The participants completed a face-to-face questionnaire administered by a trained female interviewer at a dedicated site in a private room where no identifying information was collected. Participants could refuse to answer any question. The interview lasted approximately 60 minutes, after which the participants were tested for HIV in accordance with the national guidelines using parallel rapid testing with Determine (Alere, Waltham, Massachusetts, USA) and Hexagon HIV (HUMAN Diagnostics Worldwide, Wiesbaden, Germany). All indeterminate tests, 10% of negative tests, and all positive tests were re-tested using enzyme-linked immunosorbent assays in the National Public Health Lab of The Gambia [7]. Study staff provided pre-test and optional post-test counseling, as well as referrals to government anti-retroviral therapy (ART) clinics to those who tested HIV-positive. Participants who screened positive for other adverse health outcomes including symptoms of STIs or depressive symptoms were given referrals to local health clinics and available psychological services. Data from the questionnaires were dually entered by two data entrants. A Visual Basics module in Microsoft Access was used to compare the data and reconcile discrepancies.

### Ethics

Data collection was approved by the National Scientific and Ethics Committee in The Gambia and secondary data analysis was approved by the institutional review board at Johns Hopkins School of Public Health. Per WHO ethical guidelines for violence research [45], referrals for counseling services for gender-based violence were provided through Action AID.

### Study measures

All measures were self-reported, except for HIV status, which was tested for using Determine and Hexagon HIV rapid tests. The primary exposure, *forced sex by a client*, was defined as ever having a client use force or violence to make the participant have sex or certain kinds of sex. Participant’s responses were dichotomous, (yes/no). The primary exposure for this analysis was specifically forced sex by a client as opposed to a range of sexual or physical violence variables. This exposure was chosen based on the more direct pathway between forced sex and sexual and reproductive health outcomes [46], which is the primary focus of this paper. Data on forced sex by non-client perpetrators was not collected using consistent language in this survey, using the word “rape” as opposed to “forced sex”, and was therefore not included as an exposure in this analysis. The primary exposure for this analysis is consistent with best practices when measuring sexual violence, which do not require that participants self-identify as being “raped” when measuring the prevalence of forced sex [47], as well as data showing that the primary perpetrators of sexual violence against FSW are clients [13,23,31].

Participants also self-reported demographic characteristics including age, marital status, number of children, education level (highest attained), city of origin, and the age at which they entered sex work. Age of entry was assessed by asking, “How old were you the first time you traded or sold sex or sexual acts in exchange for money, favors or goods?” The length of time that a woman has been in sex work was then calculated by subtracting the age she reported to have entered sex work from her current reported age. Participants also self-reported an additional set of violent, coercive, and otherwise potentially traumatic experiences including: if they, “had ever been beaten up as a result of selling sex”, “had ever felt verbal or physical harassment as a result of selling sex”, “had ever been tortured as a result of selling sex”, “had ever been blackmailed as a result of selling sex”, “have ever been offered more money to have non-condom protected sex” or “had ever been to jail or prison”. Primary health outcomes assessed were: *depressive symptoms,* defined as reporting a sad or depressed mood for more than 2 weeks at a time in the past 3 years; r*eceiving an STI test*, defined as having been tested for syphilis, gonorrhea, chlamydia or herpes in the past 12 months; *unwanted pregnancy*, defined as ever having a pregnancy that was unwanted at the time when the woman found out she was pregnant; and *condom availability,* defined as responding “no access” or “difficult access” or “somewhat difficult access” to condoms as opposed to reporting “somewhat easy access” or “very easy access” when asked, “Do you have access to condoms when you need them?” The question assessing depressive symptoms was not a validated scale intended to provide a clinical diagnosis, but rather an initial screen signaling the potential need for further mental health evaluation.

### Statistical analysis

Prevalence estimates of client-perpetrated forced sex were calculated overall and by demographic characteristics. Based on the type and distribution of the variable, the chi-squared test and the Wilcoxon rank-sum test were selected to evaluate the difference between groups of participants exposed and unexposed to forced sex. Descriptive analysis and bivariate logistic regression models were constructed to explore associations of forced sex by a client with other forms of abuse, as well as the available mental and physical health outcomes. Models were then adjusted for participant age, age of entry into sex work, years in sex work, and HIV status. The multivariate model for unwanted pregnancy was additionally adjusted for number of children. Possible confounders were selected based on previous literature showing the associations between these variables and the health outcomes [48-57]. Models were adjusted for *age* and *number of years in sex work* in order to control for women that may be at higher risk for negative health outcomes because they are older or have been participating in sex work longer. *Age of entry* was controlled for because earlier age of entry has been shown to be associated with higher levels of reported abuse, reporting more drug and alcohol use, as well as childhood abuse and depression as an adult [48-50]. *HIV status* was controlled for because HIV positive status has shown to be associated with depression [51,52], unwanted pregnancy [53], and increased exposure to sexual violence [54-56]. *Number of children* was controlled for in this analysis based on norms in the field where previous studies examining unwanted pregnancy control for fertility history (including the number of previous children) [56,57]. Reports of “rape” by any perpetrator did not differ significantly by group exposed and unexposed to forced sex by a client and so was not controlled for in adjusted analysis. Missing data (<4%) was dealt with through list-wise deletion. Data were analyzed using Stata statistical software 12.0 [58].

## Results

Lifetime client-perpetrated forced sex was prevalent at 29% (70/251). Participants were between the ages of 17–51 at the time of interview, with a median age of 31. Among participants in this sample, the median number of children was 1, the median age of entry into sex work was 25, and the median number of years spent in sex work at the time of the interview was 4. The participants were most likely to be widowed/divorced, 69% (173/251), followed by being single or never married, 23% (57/251). Over a third of participants, 37% (92/251), completed some or all of secondary school, 30% (75/251) completed some or all of primary school, 30% (74/251) had never attended school and 3% (6/251) had a post-high school education. The study found that 17% (40/251) of participants were living with HIV. Of those who had experienced forced sex, 19% (13/40) were HIV positive compared to 15% (26/40) of women who had not experienced forced sex [Table 1]. There were no significant differences in socio-demographic characteristics between the groups who experienced forced sex by clients compared to those who had not.

**Table 1:** **Baseline demographics by group**

	**Total sample n = 251**	**Exposed to forced sex n = 70**	**Unexposed to forced sex n = 171**	**p-value**
Forced sex: n (%)	70 (29.0)	--	--	--
HIV positive: n (%)	40 (16.8)	13 (18.6)	26 (15.2)	.52^+^
Age distribution: Median	30.0	30.5	30.0	.54^
Inter Quartile Range	26 - 35	26 - 36	26 - 35	
Median number of children	1.0	2.0	1.0	.19^
Inter Quartile Range	1 - 3	1 - 3	1 - 3	
Median age at entry into sex work in years	25.0	24.0	25.0	.43^
Inter Quartile Range	20 - 30	19 - 28	20 - 30	
Median years in sex work	4.0	4.5	4.0	.09^
Inter Quartile Range	2 - 9	2 -10	2 – 8	
Marriage status: n (%)				.27^+^
Married/Cohabitating	5 (2.0)	2 (2.9)	3 (1.8)	
Widowed/Divorced	173 (68.9)	54 (77.2)	112 (65.5)	
Single/never married	57 (22.7)	13 (18.6)	43 (25.1)	
Education level: n (%)				.86^+^
Never attended	74 (30.0)	20 (29.0)	53 (31.2)	
Some or completion of primary school	75 (30.4)	23 (33.3)	48 (28.2)	
Some or completion of secondary school	92 (37.3)	25 (36.2)	65 (38.2)	
Post high school, vocational or college	6 (2.4)	1 (1.5)	5 (2.4)	
Region of origin: n (%)				.41^+^
Kanifing municipality	30 (12.1)	12 (17.1)	16 (9.4)	
Banjul	10 (4.0)	5 (7.1)	5 (2.9)	
West Coast region	24 (9.7)	5 (7.1)	17 (10.0)	
North bank region	18 (7.3)	6 (8.6)	12 (7.1)	
Lower River region	6 (2.4)	1 (1.4)	5 (2.9)	
Upper River region	16 (6.5)	3 (4.3)	13 (7.7)	
Outside Gambia	143 (57.7)	38 (54.3)	101 (59.4)	

+p-value calculated from Pearson chi squared test.

^p-value calculated from Wilcoxon rank-sum test.

Other forms of abuse against FSW were common [Table 2]. About 1 out of 5, 21% (49/251), reported ever having been beaten up as a result of selling sex, and of these women the most common reported perpetrator was regular clients, 26% (13/49), followed by a one-time client, 20% (10/49), non-paying partner, 20% (10/49), uniformed police officer, 18% (9/49), and family member, 12% (6/49). Additionally, 40% (96/251) of the participants reported having been verbally or physically harassed as a result of selling sex, and 33% (81/251) reported having been tortured. FSW also reported high levels of coercion, with 42% (102/251) of women indicating they had been offered more money to have sex without a condom, and 25% (60/251) reporting having been blackmailed. In adjusted analyses, women who experienced forced sex by a client were significantly more likely to have experienced physical abuse as a result of selling sex (31.9% vs. 16.6% aOR: 2.44, CI: 1.22 – 4.85 p < .05), been tortured as a result of selling sex (44.3% vs. 28.8% aOR: 1.85, CI: 1.01 – 3.42 p < .05), and been blackmailed as a result of selling sex (37.1% vs. 19.9% aOR: 2.33, CI: 1.22 – 4.47 p < .05) [Table 2].

**Table 2:** **Associations of forced sex by a client (FS) with other forms of violence and health outcomes**

	**Total sample: n (%) n = 251**	**Exposed to FS: n (%) n =70**	**Unexposed to FS: n (%) n = 171**	**Crude OR (95% CI)**	**P-value**	**Adjusted OR^ (95% CI)**	**P-value**
**Violence Outcomes:**							
Ever beaten up as a result of selling sex	49 (21.1)	22 (31.9)	27 (16.6)	2.36 (1.22 - 4.53)	.01*	2.44 (1.22 - 4.85)	.01*
By regular client	13 (26.5)						
By one-time client	10 (20.4)						
By non- paying partner	10 (20.4)						
By uniformed officer	9 (18.4)						
By family member	6 (12.2)						
Ever verbally or physically harassed as a result of selling sex	96 (38.5)	31 (44.3)	64 (37.4)	1.33 (.76 - 2.34)	.32	1.25 (.70 - 2.26)	.45
Ever tortured as a result of selling sex	81 (32.7)	31 (44.3)	49 (28.8)	1.96 (1.10 - 3.49)	.02*	1.85 (.99 - 3.42)	.05*
Ever blackmailed as a result of selling sex	60 (24.5)	26 (37.1)	34 (19.9)	2.38 (1.30 - 4.40)	<.01*	2.33 (1.22 - 4.47)	.01*
Ever offered more money to have sex without a condom	102 (42.1)	30 (42.9)	72 (42.9)	1.00 (.57 - 1.76)	1.0	.96 (.53 - 1.73)	.89
Ever jailed	11 (4.8)	6 (9.5)	5 (3.1)	3.24 (.95 - 11.04)	.06	2.29 (.62- 8.50)	.21
**Health Outcomes:**							
Depressive Symptoms~	154 (62.6)	51 (73.9)	100 (58.8)	1.98 (1.06 - 3.68)	.03*	2.15^ (1.10 - 4.16)	.02*
STI test in the past 12 months	112 (45.3)	23 (32.9)	84 (49.7)	.49 (.28 - .89)	.02*	.49^ (.26 - .91)	.02*
Unwanted pregnancy (Over lifetime)	54 (23.8)	19 (29.2)	35 (22.6)	1.42 (.74 - 2.72)	.29	2.69 > (1.12 - 6.49)	.03*
Difficult or no access to condoms^+^	121 (48.9)	48 (68.6)	71 (42.0)	3.01 (1.67 - 5.43)	<.001*	3.32^ (1.75 - 6.26)	<.001*

*p-value significant at .05 level.

~Screen for depressive symptoms through the question “Have you felt sad or had a depressed mood for more than two weeks at a time in the last three years”.

^+^Variable coded dichotomously comparing “no”, “difficult”, or “somewhat difficult” access compared to “very easy” or “somewhat easy” access to condoms.

^Adjusted for age, age of entry into sex work, years spent in sex work, and HIV status.

>Adjusted for age, age of entry into sex work, years spent in sex work, HIV status, and number of childre006E.

In analyses examining the association between health outcomes and forced sex by a client, women reporting forced sex were significantly more likely to suffer from symptoms of depression (73.9% vs. 58.8%, OR: 1.98 CI: 1.06 – 3.68 p < .05, aOR: 2.15 CI: 1.10 – 4.16 p < .05), to report “no”, “difficult” or “somewhat difficult” access to condoms (68.6% vs. 42.0% OR: 3.01 CI: 1.67 – 5.43 p < .001, aOR: 3.32 CI: 1.75 – 6.26 p < .001), and report an unwanted pregnancy (29.2% vs. 22.6%, OR: 1.42 CI: .74 – 2.72 p = .29, aOR: 2.69 CI: 1.12 – 6.49 p < .05) after adjusted analysis. Participants who reported forced sex from a client were also significantly less likely to have an STI test in the past 12 months (32.9% vs. 49.7% OR: .49 CI: .26 - .91 p < .05, aOR: .49 CI: .26 - .91 p < .05) [Table 2].

## Discussion

These findings demonstrate extensive client-perpetrated sexual violence against FSW in The Gambia, with over one in four women (29%) reporting having been forced to have sex with a client in their lifetime. This analysis adds to a growing body of research demonstrating the negative health impacts of sexual violence against FSW. Women who had been forced to have sex by a client were significantly more likely to be victims of other violent crimes such as being beaten up or tortured, which may further increase their vulnerability for negative health outcomes.

Victimization was significantly associated with unwanted pregnancy. Sexual violence can impart risk for unintended pregnancy directly, as well as put survivors on a trajectory of limited control over condom negotiation and use in future sexual encounters [20,26]. The significant association between sexual violence and unwanted pregnancies among FSW in The Gambia is potentially contextualized by previous research among FSW, which showed that condoms are often not used during incidents of forced sex [12,22,23], and is consistent with previous research showing that victims of forced sex report high percentages of unwanted pregnancy and abortion [35,59,60]. Together, these findings call for a more integrated response to sexual violence in The Gambia for survivors of forced sex, including access to emergency contraceptives (EC).

Ongoing initiatives in The Gambia to address sexual violence include a national action plan and development of a communication strategy on gender-based violence. The Gambia recently reaffirmed its commitment to confronting sexual violence by approving the Sexual Offences Bill of 2013, which seeks to punish offenders for sexual violence, harassment, and other threatening behavior [61]. However, the practical implementation of the bill has not been studied and there continues to be limited guidelines for service provision for survivors of sexual violence. The evidence suggests that providing services to survivors of violence, including access to EC, could prevent unwanted pregnancies and abortions, both of which may have negative health consequences for Gambian women, especially in contexts where abortion is illegal and women may be seeking abortion services in unsafe environments [44].

Findings from this study show high levels of depressive symptoms among FSW in The Gambia (62.6%). Previous research, using a modified Edinburgh Depression Scale, has shown that approximately 10.3% of women of reproductive age in the general population in The Gambia reported depressive symptoms that suggest a substantial probability of depression [62]. While these findings are not directly comparable to the current study, given the use of a different screening question for depressive symptoms, this suggests that the prevalence of depressive symptoms may be elevated among FSW compared to women in the general population. Current findings highlight FSW’s vulnerability for poor mental health outcomes and the need for the availability of mental health services for this population. In addition, forced sex is associated with depressive symptoms, which calls attention to the role that sexual violence may play in mental health outcomes for FSW. These findings advance earlier evidence that violence affects the mental health of women in general [63,64] as well as FSW, who are estimated to be two to five times more likely to report depression if they have experienced forced sex compared to women who did not report forced sex [25,42]. The observed prevalence of depressive symptoms strengthens the call of past research for mental health services for survivors of violence. In Nepal, researchers found that depression among FSW was also associated with HIV risk behavior, emphasizing the need to address mental health issues among FSW as part of a comprehensive HIV prevention package [42].

In The Gambia there is limited research on mental health issues and limited infrastructure for mental health treatment, including lack of human resources and budget allocations for services [65]. The Gambia’s Ministry of Health and Social Welfare collaborated with the WHO and finalized a strategic plan to improve the treatment gap in mental health services in 2006 [66]. The major objectives of this plan were to provide accessible mental health and substance abuse services, and protect the rights of people with mental and substance use disorders. This plan could be strengthened by including a focus on addressing the mental health needs of FSW and survivors of sexual violence at the community level. This could include supporting the efforts of local organizations to build the capacity of clinical and non-clinical staff to respond to survivors’ needs, engage in community sensitization efforts surrounding mental health disorders, FSW, and violence, as well as increase resource allocation for improved service delivery. Research in The Gambia, from 2011, has suggested that providing medication for depression and other mental health disorders may not be a feasible immediate strategy to treat depression, given the current cost-barriers and absence of prescribers, which greatly limits widespread use [67]. Intermediate strategies to support FSW and survivors of violence in The Gambia could include psychotherapeutic support groups, which has shown some promise as a depression intervention among HIV positive adults in Uganda [68], sociotherapy to build social bonds, and group interpersonal therapy which have both shown positive results in responding to the mental health needs of survivors of violence in Rwanda [69] and rural Uganda [70]. FSW-specific research must be conducted to learn if these interventions show promise for FSW populations.

Current evidence suggests that sexual violence victimization at the hands of clients is also associated with lower access to condoms and decreased likelihood of receiving an STI test in the past 12 months. These findings point to an unmet need in sexual health services among FSW who are survivors of violence. Qualitative research has suggested that in situations where FSW have immediate emergencies that threaten their safety, such as violence and abuse, they are less likely to focus on relatively longer-term issues of HIV, STIs, and pregnancy [71]. This may explain why women who report having experienced forced sex also report having less access to condoms and are less likely to be tested for STIs; in other words, crisis situations may undermine their ability to plan and control their sexual health. Responding to the unmet need for sexual health services through outreach to FSW could be a potential strategy to reach the women who have less access to services, including survivors of violence. Reaching these women, who are most vulnerable for poor health outcomes, is an important strategy to improve health and wellbeing for Gambian FSW.

### Limitations

A limitation of this cross-sectional design is that the direction of the associations cannot be determined, and while forced sex is associated with various health outcomes, causality cannot be shown. The sample for this study was relatively small and recorded a small number of events, which resulted in large confidence intervals. The sample was also derived from chain-referral and venue-based convenience sampling due to the difficulty with recruitment among this extremely mobile and vulnerable population in The Gambia. Due to non-randomized nature of this recruitment method, it cannot be determined if this sample is fully representative of FSW in The Gambia. Furthermore, data on what individuals were recruited by whom or from where were not recorded, so that data analysis adjusted for clustering by recruitment chain or venue was not possible. Future studies could address these difficulties by employing Respondent Driven Sampling (RDS) as a recruitment method. RDS was not used in the present study due to time and budget restrictions.

The current analysis, which examines forced sex perpetrated by clients, does not include forced sex by other perpetrators. The measure may therefore underestimate the prevalence of forced sex overall among FSW because it does not reflect victimization from non-paying partners, family members, police, strangers and others. However, client violence is the predominant type of sexual violence reported by FSW in many global settings [13,23,31], and is an important exposure to study in its own right, as it may have different associations with health outcomes than sexual violence at the hands of other perpetrators [72]. A surprising result was that one third of participants recorded having been “tortured as a result of selling sex”. Future research is needed to determine what abuses women are referring to when participants report having been tortured in this context. In addition, this questionnaire was intended to measure depressive symptoms and cannot be used to quantify the prevalence of clinical depression among FSW. Sexual violence indicators were reported in face-to-face interviews where participants may have been uncomfortable disclosing victimization. However, the anonymous nature of the interview, training of female interviewers to be sensitive, and participant’s ability to refuse to answer any question may have helped to mitigate under-reporting.

## Conclusions

These data provide valuable insights into the prevalence of, and health outcomes associated with, client-perpetrated sexual violence among FSW in The Gambia. More research is needed in The Gambia to examine the prevalence and correlates of forced sex against FSW by non-clients as well as sexual violence in the general population. Current findings point to an unmet need for health services for FSW survivors of sexual violence. Responding to the physical and mental health needs of survivors in The Gambia, including outreach to FSW, is a priority to improve FSW and survivor health more generally. A response to sexual violence victimization that includes the availability of emergency contraceptives, post-exposure prophylaxis, and mental health crisis services, could better meet the post-assault health needs of Gambian FSW as well as women in the general population.

Efforts to reduce sexual violence against sex workers should be prioritized in The Gambia as a way to improve the health and safety of FSW as well as address the spread of HIV/STIs. HIV/STI prevention programs often employ individual-level behavior interventions that address individual acquisition and transmission of sexually transmitted infections. However, in the cases of forced sex where women may be unable to practice preventive behaviors, interventions that target individual-level HIV risk factors such as condom use can be less potent. Programs and policies that address violence and the larger contextual issues that contribute to unsafe sex and inconsistent condom use may be more effective [73]. Recent work that modeled HIV incidence rates based on data from Kenya estimated that if violence against FSW could be reduced to 2.4%, compared to the current prevalence of 32%, that new HIV infections could be decreased by 27% among FSW, and 12% among the general adult population [74]. This work quantifies the potential impact that violence reduction among FSW can have on national HIV infection rates in an African context. The high rate of sexual violence among FSW is not only an issue of human rights and safety but also an issue of sexual, reproductive, and mental health. The Gambia’s National Reproductive Health Policy Plan (2007–2014) emphasizes the need to reduce sexual violence and ensure proper support for victims to improve reproductive health outcomes of women, but FSW are not mentioned specifically in these objectives [75]. Given the unique needs of FSW and their exposure to additional forms of violence, specifically client violence, suggests that including strategies to reduce sexual violence against FSW in The Gambia’s strategic plan would be an important step in improving sexual, physical, and mental health outcomes for Gambian sex workers and the population more broadly.
